# Leptomeningeal metastasis from non-small cell lung cancer— a *post-hoc* analysis from four randomised clinical trials

**DOI:** 10.3332/ecancer.2022.1414

**Published:** 2022-06-16

**Authors:** Vijay Patil, Vanita Noronha, Dilip Harindran Vallathol, Nandini Menon, Abhishek Mahajan, Amit Janu, Nilendu Purandare, Kumar Prabhash

**Affiliations:** 1 Department of Medical Oncology, Tata Memorial Centre, HBNI, Mumbai 400012, India; 2 Department of Radiology, Tata Memorial Centre, HBNI, Mumbai 400012, India; 3 Department of Nuclear Medicine, Tata Memorial Centre, HBNI, Mumbai 400012, India; † Both contributed equally.

**Keywords:** leptomeningeal metastasis, non-small cell lung cancer, incidence, survival

## Abstract

**Background:**

Leptomeningeal metastasis (LMM) from non-small cell lung cancer (NSCLC) is often an underdiagnosed entity, has a dismal prognosis and has very limited data from low- and middle-income countries.

**Methods:**

A single-centre study, which included 1148 adult patients diagnosed as NSCLC, with Eastern Oncology Cooperative Group *performance status* 0–2, as identified from data of four prospective randomised controlled trials. Two trials included patients who had epidermal growth factor sensitive mutations (CTRI/2015/08/006113 and CTRI/2016/08/007149) and the other two included squamous cell carcinoma (CTRI/2013/02/003422) and adenocarcinoma patients (CTRI/2014/08/00484). The key objectives were to estimate the incidence, risk factors, time to development and outcomes for LMM.

**Results:**

Out of 1148 patients, 36 patients (0.031%; 95%CI: 0.022–0.043) developed leptomeningeal metastasis. In these patients, median time to development of LM was 14.92 months (interquartile range: 7.7–21.84). Among the tested factors, the presence of brain metastasis was the only statistically significant risk factor associated with the development of LMM (*p*-value = 0.035). The median overall survival (OS) after the development of LM was 61 days (95%CI: 38.95–83.05). The median OS in driver mutated patients was 66 days (95% CI: 14.74–117.26) versus 51 days (95% CI: 14.5–87.5) (*p*-value = 0.201) in non-driver mutated patients. Only 6 (19.4%) out of 31 epidermal growth factor receptor-mutated patients received osimertinib. Patients treated with osimertinib had a median OS of 245 days (95% CI: 215.48–274.52) versus 52 days (95% CI: 22.62–81.38) for those without (*p*-value = 0.327).

**Conclusion:**

The incidence of LMM is low in the Indian population. In our study, there was no single factor which impacted survival in patients who developed LMM. This suggests that the overall prognosis is poor in patients with LMM where access to newer therapeutic modalities is limited.

## Introduction

Leptomeningeal metastasis (LMM) is a rare and underdiagnosed entity in extracranial solid tumours [1, 2]. It often occurs in the setting of a progression of systemic disease. The estimated incidence of LMM in solid tumours (excluding central nervous system tumours) is around 5%–10% [3]. The treatment of LMM has also evolved, but the prognosis of LMM remains dismal when compared to other sites of metastasis [4]. The two commonest extracranial solid tumour sites leading to LMM are breast carcinoma and non-small cell lung cancer (NSCLC) [3]. The improvement in diagnostic modalities and newer modalities of treatment of malignancies has led to an increase in the diagnosis of LMM [5, 6].

The occurrence of LMM is particularly increased in patients with driver mutations as a result of their improved outcomes with targeted therapies and better extracranial disease control [5, 6]. Previously reported studies by Yang *et al* [6] and Liao *et al* [7] in a similar setting showed higher rates of LMM among epidermal growth factor receptor (EGFR)-mutated patients when compared to mutation negative patients. Yang *et al* [6] showed a 9.4% incidence of LMM among EGFR-mutated patients when compared to 1.7% in negative ones; Liao *et al* [7] reported 212 patients (3.8%) with LMM in NSCLC of which 75 patients harboured EGFR mutations. The mechanisms responsible for the higher frequency of LMM in patients with driver mutations may be multifactorial. Longer survival and insufficient penetration of tyrosine kinase inhibitors (TKIs) into the CSF are likely to be the main explanations. With the application of TKIs, overall survival (OS) of metastatic NSCLC with driver mutations was improved in comparison to the OS of patients with wild-type. Moreover, the ratio of the CSF to plasma concentration of TKIs is low on account of incomplete penetration of the blood–brain barrier [8]. Hence, insufficient levels of TKIs, which result in continuing lower stimulation of tumour cells, may favour resistance and pave the way for LMM. Even though these are postulated, the exact mechanism has not yet been elucidated. Globally, the improvement in treatment of malignancies, with the entry of novel therapies and higher generation of targeted agents with better central nervous system penetration, has improved the median survival in LMM from 1–3 months to 3–11 months [5, 6]. However, access to these novel agents is limited in low- and middle-income countries (LMICs). There is very limited literature regarding the incidence, presentation symptoms, diagnostic modalities, treatment and outcomes of LMM with these novel therapies from LMICs. Over the last 5 years, we have reported four randomised studies in NSCLC from India [9–12]. These studies have contributed towards the improvement in outcomes of NSCLC. In view of the limited data on LMM from NSCLC from LMIC, we carried out this *post-hoc* analysis on the data obtained from these four prospective randomised studies, with a key objective to estimate the median OS in patients who develop LMM after diagnosis of NSCLC. The other objectives were to estimate the incidence of LMM, risk factors, clinical presentation and pattern of treatment.

## Materials and methods

### Patient selection

This was a *post-hoc* analysis on the data collected from the four prospective randomised controlled trials conducted on NSCLC patients at a single centre in the last 10 years. All the four studies have been published and the detailed study protocols are available online.

These trials included the following:

1. Patients who had EGFR sensitising mutations enrolled in a 1:1 fashion either in the pemetrexed versus gefitinib trial (Pem-Gef study, February 2012 and April 2016) [9] or gefitinib versus gefitinib plus pemetrexed and carboplatin trial (Gef versus Gef+C study, August 2016 and August 2018) [10]. The primary end point was progression-free survival (PFS) with OS being a key secondary end point in both studies.
2. Patients who were diagnosed with squamous cell carcinoma of the lungs were enrolled in a 1:1 fashion in the low-dose gemcitabine with platinum versus standard-dose gemcitabine with the platinum trial (May 2013–March 2018) [11]. OS was the primary end point.
3. Patients with adenocarcinoma who were enrolled in the pemetrexed versus erlotinib maintenance trial (November 2014–March 2017) with quality of life being the primary end point [12].

### Data collection

The data were extracted from the trial database of the four aforementioned prospective trials. The demographic details at enrollment in the trial and at LMM, driver mutation status, brain metastasis status and treatment details were extracted. In addition, clinical presentation, diagnostic details, treatment, response assessment and the outcomes for LMM were extracted.

### Outcome variable

The occurrence of LMM was defined as positive cerebrospinal fluid (CSF) cytology or CSF sampling performed only when symptomatic. Contrast-enhanced magnetic resonance imaging (MRI) with gadolinium contrast features was consistent with the diagnosis of LMM [11]. CSF cytology was carried out in 17 patients and MRI was carried out in 27 patients. CSF cytology was especially carried out in all symptomatic patients who were eligible for intrathecal therapy. These neuroimaging features were the unequivocal presence of leptomeningeal nodules or enhancement. The response to LMM was assessed in accordance with the RANO working group proposal [12]. OS was defined as the time in months from the date of diagnosis of LMM to the date of death.

### Statistical analysis

SPSS version 17 and RStudio version 3.1 were used for analysis. Descriptive statistics were carried out. The incidence of LMM was calculated using a competing risk analysis. Death due to any other cause before developing LMM was considered as competing. The factors affecting LMM development were identified using binary logistic regression analysis. The factors tested were age, performance status on the Eastern Oncology Cooperative Group (ECOG PS) scale, driver mutation status and previous brain metastasis. OS was estimated using the Kaplan–Meier analysis. COX regression analysis was used to identify factors affecting OS. *p*-value of 0.05 was considered significant.

## Results

### Incidence of LMM

**Figure 1.**
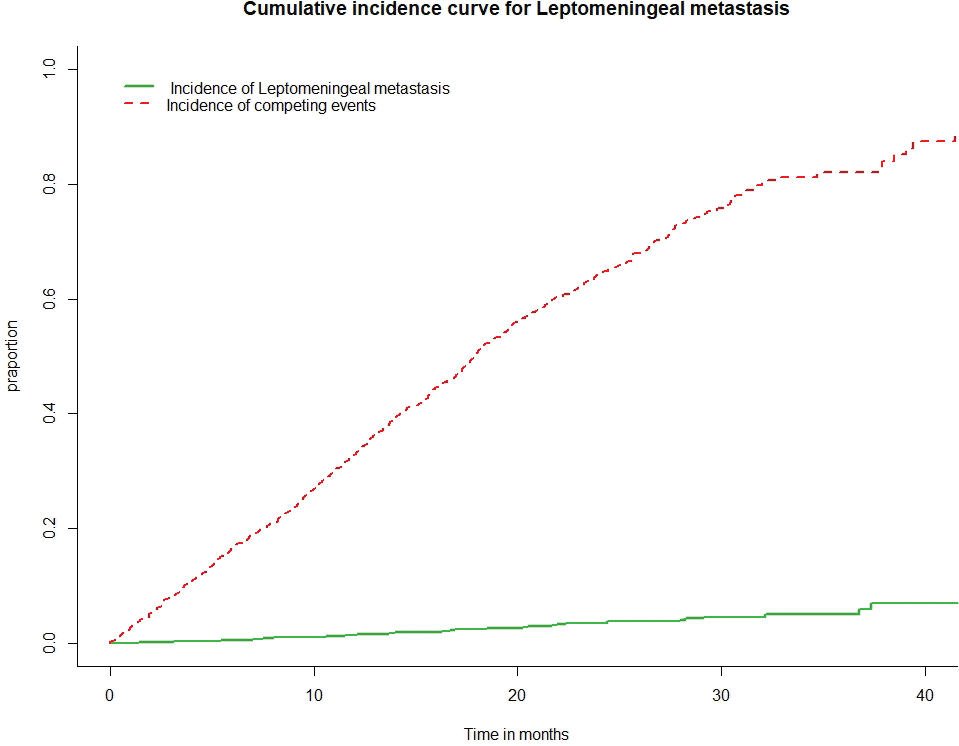
Incidence of leptomeningeal metastasis in the whole cohort. The green line depicts the incidence of leptomeningeal metastasis and the red line depicts the occurrence of a competing event – death.

From our database of 1148 patients, we identified 36 (0.031%; 95%CI: 0.022–0.043) patients of NSCLC who developed LMM in the past decade. The incidence of LMM is shown in Figure 1. The median time to develop LMM was 14.92 months (interquartile range (IQR): 7.7–21.84). The risk factors for development of LMM are shown in Table 1.

### Baseline characteristics in patients with LMM

Data on baseline patient characteristics at development of LMM are given in Table 2. There were 22 males (61.1%) and 14 females (38.9%) in the analysis. There were 23 patients (63.9%) who were below the age of 60 (non-elderly group) and 13 (36.1%) were ≥60 years (elderly group). Twenty-four of the 36 patients (66.7%) had developed LMM after one line of therapy, while 6 patients (16.7%) received 3 or more lines of therapy.

### Clinical features

Data on symptoms at presentation were available for 31 out of 36 patients (86.1%). Majority of the patients (15 of the 31 evaluable patients (48.4%)) presented with symptoms of altered higher mental functions or seizures. Full data on symptoms at presentation is presented in Table 3. The diagnosis of LMM was made by CSF analysis alone in 10 patients (27.8%), while imaging was performed in 18 patients (50%). Diagnosis by both CSF analysis and imaging was made in 8 patients (22.2%). Only 1 of the 18 patients (5.5%) who were diagnosed by CSF analysis required 2 lumbar punctures. Systemic progression was present in 20 out of 36 patients (55.6%) of NSCLC at the time of diagnosis of LMM out of which 10 patients (20%) had brain progression. The median time for the development of LMM was 14.92 months (IQR: 7.7–21.84). Out of the 36 patients, 10 patients (27.8%) had brain progression or new-onset brain metastasis. The details of the diagnosis of LMM are incorporated and presented in Table 3.

### Treatment

Twenty-three patients out of the 36 (63.9%) patients with leptomeningeal metastasis received some form of treatment. Details are presented in Table 3. Out of the 36 patients, 6 received chemotherapy (16.7%) for leptomeningeal progression. Eighteen out of 31 EGFR mutation-positive patients (58.1%) received first-generation TKIs; 7 received high-dose TKI (erlotinib/gefitinib) (38.9%) and the rest standard dose (61.1%). Only 6 (19.4%) out of 31 EGFR-mutated patients received osimertinib. Intrathecal treatment was administered to 11 of the 36 patients (30.6%) of which 4 received triple intrathecal therapy (36.4%) (methotrexate, cytarabine and hydrocortisone) and 7 received single-agent methotrexate (63.6%). Patients with objective responses were treated with osimertinib either with standard-dose or high-dose intrathecal therapy. Patients with a stable disease were commonly treated with a combination of intrathecal therapy, TKI (high-dose erlotinib or gefitinib) and chemotherapy (most commonly combining TKI and intrathecal therapy). The details of the responses to intrathecal therapy are highlighted in Table 4.

### Response

Response assessment was conducted in all 23 patients (63.9%) who were treated. Response was seen in 3 patients (13%), stable disease in 11 (47.8%) and in the remaining 9 (39.2%) had a progressive or refractory disease.

### Overall survival

**Figure 2.**
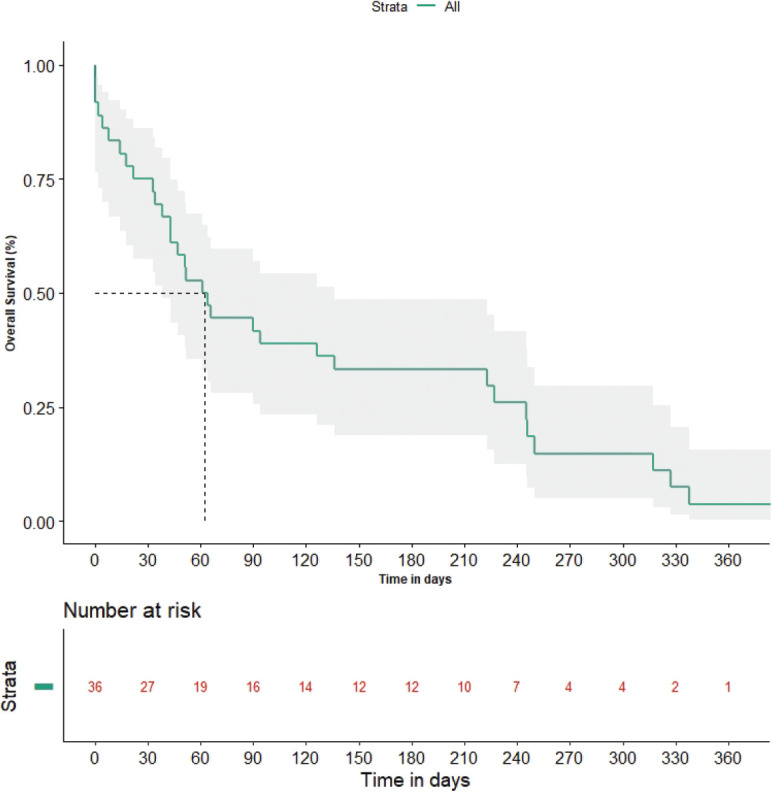
OS graph. OS in days with its 95% confidence interval is depicted. The median OS value is depicted with dashed lines.

The median OS after development of LM was 61 days (95% CI: 38.95–83.05) (Figure 2). None of the tested factors, including age (Hazard ratio = 0.829; 95% CI: 0.383–1.792, *p*-value = 0.633), ECOG PS (Hazard ratio = 0.745; 95% CI: 0.306–1.813, *p*-value = 0.517), driver mutation status (Hazard ratio = 2.188, 95% CI: 0.756–6.336, *p*-value = 0.149), presence of brain metastasis (Hazard ratio = 1.432, 95% CI: 0.609–3.365, *p*-value = 0.411) or presence of positive CSF cytology (Hazard ratio = 1.026; 95% CI: 0.455–2.315, *p-*value = 0.95), had an impact on OS. The median OS in driver mutated patients was 66 days (95% CI: 14.74–117.26) versus 51 days (95% CI: 14.5–87.5) (*p*-value = 0.201) in non-driver mutated patients. Among the driver mutated patients, patients treated with osimertinib had a median OS of 245 days (95% CI: 215.48–274.52) versus 52 days (95% CI: 22.62–81.38) (*p*-value = 0.327) for those without.

### Discussion

LMM is a devastating complication associated with solid malignancies with limited treatment options and poor OS. To the best of authors’ knowledge, this is one of the few studies that has retrospectively looked into four prospective single-centre trials on LMM in NSCLC. The incidence of LMM in the current study is 0.03%. This is much lower than the incidence reported in multiple studies which ranges between 3% and 9% [4, 5]. The question is why was the incidence of leptomeningeal metastasis low in our study. The reason may be manifold. Firstly, evaluation of the craniospinal axis was not carried out routinely. It was performed by physicians only when brain metastasis or LMM were suspected. Hence, asymptomatic LMM was missed. This would have led to falsely low rates of LMM. Secondly, we used strict criteria for diagnosis of LMM which were either cytological positivity or radiological evidence. The diagnosis of LMM based on biochemical or clinical parameters was avoided even though these recommendations are provided by the NCCN and the ESMO-EANO group [13, 14]. Thirdly, access to second and third-generation TKIs and immunotherapy was restricted in our studies. Therefore, the 18-month OS rate in EGFR mutation-positive patients in India is between 48.7 and 74.3% [9, 10] as opposed to the 18-month OS rate with third-generation TKIs of 85% [15, 16]. Probably because of this, patients died due to extracranial progression (competing causes), thus limiting the probability of progression to LMM. Fourthly, our data were from a prospective cohort of four studies. As opposed to this, the incidence data in most of the studies has come from retrospective studies with its likelihood of selection bias. Lastly, there might be a lower occurrence of LMM in Indian patients.

Time to develop LMM in the current study was 14.92 months. This is similar to the time to development seen in other studies. In a retrospective analysis from Kuiper *et al* [17], time to development of leptomeningeal metastasis was 13·3 months. In our study, the trend of occurrence of LMM was more in driver mutation-driven patients, which is in concurrence with previous studies. In a study by Li *et al* [6], the incidence of LMM was found to be 9.4% in patients with EGFR mutations versus 0.7% in wild-type EGFR patients (*p* < 0·001). The higher occurrence of LMM in patients with brain metastasis suggests that it is important to consider the occurrence of LMM in patients with brain metastasis on follow-up [18].

The clinical features seen in our study had a predominance (nearly half) of higher mental function disturbance and seizures. This is in line with the data published by Pan et al where adenocarcinoma-related LMM mainly leads to higher mental function symptoms [19]. Another interesting aspect of the data was presentation of LMM with cerebrovascular accident-like features. This suggests that LMM probably should be a provisional diagnosis when CVA-like symptoms are seen in NSCLC patients.

Nearly 40% of the patients in the current study did not receive cancer-directed treatment at LMM. This is in line with the data across the world [20]. The outcomes of patients with poor performance status, fixed extensive neurological deficits or with extracranial progression are dismal [21]. Hence, patients with these features are not treated routinely at our centre. The variable use of intrathecal therapies with systemic therapies seen in the study is in concurrence with the limited efficacy of intrathecal therapies seen in the literature [1, 22]. There is no evidence of improvement in OS or PFS with the use of intrathecal therapies [1, 22]. The median OS after development of LMM was around 2 months, which is consistent with that quoted in the literature [21–23]. As per our study, the use of intrathecal therapy had some form of response among patients as compared to those who did not receive intrathecal therapy.

Our results are inferior to those reported in studies focussing on novel agents in driver mutated patients. The recently published BLOOM study has shown promising results with the use of high-dose osimertinib (80 mg BID) in EGFR-mutated NSCLC with LMM [25]. In our study also there was a trend towards higher OS in patients receiving osimertinib. However, the numbers were few (6 out of 31) to have adequate power for a statistical analysis. Thus, our data along with the literature suggests that non-driver mutated patients have poor prognosis and even driver mutated patients would have similar prognosis if exposure to third-generation novel agents is not possible. The outcomes of EGFR-mutated NSCLC treated with first-generation tyrosine kinase inhibitors can be improved by administering either VEGF inhibitors [26] or with the addition of chemotherapy [10] or alternatively by substituting it with novel third-generation agents [18]. Moreover, the results imply that in patients with LMM, the use of agents like osimertinib is warranted.

The study limitations are that it was carried out among the Indian population and in a single centre. Access to third-generation therapies and immunotherapy was limited. However, this is also a strength as it is a pointer towards the likely outcomes in LMICs where access to these agents are limited. The strength of the study is the strict criteria used for LMM diagnosis and the radiology review for confirmation of the presence of LMM.

## Conclusion

The incidence of LMM is low in the Indian population. In our study, there was no single factor which impacted survival in patients who developed LMM. This suggests that the overall prognosis is poor in patients with LMM where access to newer therapeutic modalities is limited.

## Funding

No funding was received for this study.

## Conflicts of interest

The authors declare that they have no conflicts of interest.

## Authors’ contributions

Vijay Patil and Kumar Prabhash – concept, design, execution, data collection, analysis, interpretation, first draft and final draft.

Vanita Noronha – execution, data collection, interpretation, first draft and final draft.

Dilip Harindran Vallathol – concept, data collection, interpretation, first draft and final draft.

Nandini Menon, Abhishek Mahajan, Amit Janu and Nilendu Purandare – execution, data collection and final draft

## Figures and Tables

**Table 1.** Factors predicting the development of leptomeningeal metastasis.

Variable	Variable factors	Leptomeningeal metastasis (%)	OR (95%CI)	*p*-value
Age	Elderly (*n* = 439)	12 (2.7)	0.89 (0.44–1.8)	0.745
	Non-elderly (*n* = 709)	24 (3.4)	Reference	
Gender	Male (*n* = 744)	23 (3.1)	0.567 (0.283–1.135)	0.109
	Female (*n* = 404)	13 (3.2)	Reference	
ECOG PS	ECOG PS 0-1 (*n* = 1,033)	30 (2.9)	0.674 (0.269–1.687)	0.399
	ECOG PS 2 (*n* = 115)	8 (5.2)	Reference	
Histopathology	Non-squamous (*n* = 858)	35 (4.1%)	2.731 (0.32–23.318)	0.359
	Squamous (*n* = 290)	1 (0.3%)	Reference	
Driver mutation status	Present (*n* = 658)	31 (4.7)	Reference	0.064
	Absent (*n* = 490)	5 (1)	0.379 (0.136–1.058)	
Brain metastasis	Present (*n* = 153)	10 (6.5)	Reference	0.035
	Absent (*n* = 995)	26 (2.6)	0.45 (0.214–0.946)	

ECOG PS, Eastern Oncology Cooperative Group performance status; OR, odds ratio; CI, confidence interval.

**Table 2.** Patient characteristics.

Variable	Value (*n* = 36)
Age Elderly Non-Elderly	13 (36%) 23 (64%)
Gender Male Female	22 (61%) 14 (39%)
ECOG PS 0–1 2–3	29 (81%) 7 (19%)
Histology Squamous Non-squamous	1 (2.8%) 35 (97.2%)
Driver Mutation EGFR mutation Exon 19 deletion Exon 21 L858R Others	31 (86.1%) 19 (61.3%) 10 (32.3%) 2 ( 6.4%)
Brain metastasis Yes No	9 (25%) 27 (75%)

**Table 3.** Clinical features, diagnosis and treatment details.

Variable	Value (*n* = 36)
Symptoms Altered higher mental functions(memory defects/altered sensorium) and seizures Cerebrovascular accident like episodes/visual disturbances Headache and dizziness	15 (48.4%) 6 (19.4%) 10 (32.2%)
Diagnosis CSF analysis Imaging CSF analysis and Imaging	10 (27.8%) 18 (50%) 8 (22.2%)
Number of other sites of metastasis 1 2 3	5 (13.8%) 21(58.3%) 10 (27.8%)
Brain metastasis prior to leptomeningeal metastasis Yes No	9 (25%) 27 (75%)
Number of previous lines of therapy 1 2 3	24 (66.6%) 6 (16.7%) 6 (16.7%)
Brain metastasis or progression at the time of leptomeningeal metastasis Yes No	10 (27.8%) 26 (72.2%)
Intrathecal therapy Methotrexate Triple intrathecal therapy	11 (47.8%) 7 (63.6%) 4 (36.45)
Chemotherapy Pemetrexed Docetaxel Paclitaxel	6 (26%) 2 (33.3%) 3 (50%) 1 (16.7%)
Tyrosine kinase inhibitor Erlotinib High Standard Gefitinib High Standard Osimertinib High Standard	18 (78.2%) 3 (16.7%) - 3 (33.3%) 6 (66.7%) 1 (16.7%) 5 (83.3%)

**Table 4.** Responses in patients receiving intrathecal therapy.

Response	Number of patients	Intrathecal therapy (Number of patients)
Complete response	1	Intrathecal methotrexate (1)
Partial response	2	Intrathecal therapy (2) Triple intrathecal therapy (1) Intrathecal methotrexate (1)
Stable disease	11	Intrathecal therapy (6) Triple intrathecal therapy (2) Intrathecal methotrexate (4) No intrathecal therapy (5)
Progressive disease	17	Intrathecal therapy (2) Triple intrathecal therapy (0) Intrathecal methotrexate (2) No intrathecal therapy (15)
